# Contrast-enhanced CT-based deep learning model assists in preoperative risk classification of thymic epithelial tumors

**DOI:** 10.3389/fonc.2025.1616816

**Published:** 2025-07-31

**Authors:** Xuhui Zhao, Lingyu Zhang, Li Liang, Qi Zhang, Wencan Wang, Junlin Li, Hua Zhang, Chunhai Yu, Lingjie Wang

**Affiliations:** ^1^ Department of Medical Imaging, Shanxi Provincial People’s Hospital Affiliated to Shanxi Medical University, Taiyuan, Shanxi, China; ^2^ Department of Medical Imaging, First Hospital of Shanxi Medical University, Taiyuan, Shanxi, China; ^3^ Department of Medical Imaging, Inner Mongolia People’s Hospital, Hohhot, Inner Mongolia, China; ^4^ Department of Medical Imaging, Shanxi Cancer Hospital, Shanxi Hospital Affiliated to Cancer Hospital, Chinese Academy of Medical Sciences, Cancer Hospital Affiliated to Shanxi Medical University, Taiyuan, Shanxi, China

**Keywords:** deep learning, convolutional neural network (CNN), radiomics, thymic epithelial tumors, computed tomography

## Abstract

**Background:**

This study aimed to develop and evaluate a deep learning (DL) model utilizing contrast-enhanced computed tomography (CT) to assist radiologists in accurately stratifying the risk of thymic epithelial tumors (TETs) based on the World Health Organization (WHO) classification.

**Methods:**

Involved retrospectively enrolling clinical data from 266 patients with histopathologically confirmed TETs from two centers: Center 1 (training set, n=205) and Center 2 (external testing set, n=61). Six DL models (DenseNet 121, ResNet 101, Inception V3, VGG 11, MobileNet V2, and ShuffleNet V2) were developed and evaluated using venous-phase CT images, alongside a traditional radiomic model using a support vector machine (SVM) for comparison. Diagnostic performance of junior and senior radiologists in distinguishing low-risk thymoma (LRT) from high-risk thymoma (HRT) was assessed with and without the assistance of the optimal DL model.

**Results:**

The ResNet 101 model emerged as the best performer among six DL models, achieving an AUC of 0.876, accuracy of 0.820, sensitivity of 0.878, specificity of 0.700, positive predictive value of 0.857, and negative predictive value of 0.737 in the external testing set, outperforming the traditional radiomic model (AUC, *p* < 0.05). Notably, DL model significantly improved junior radiologists’ diagnostic performance, with an average AUC of 0.822, approaching senior radiologists’ average AUC of 0.859 (*p* > 0.05).

**Conclusions:**

This study demonstrated that a DL model based on contrast-enhanced CT can reliably assist radiologists in preoperative risk stratification of TETs, bridging the diagnostic performance gap between junior and senior radiologists and supporting clinical decision-making.

## Introduction

Thymic epithelial tumors (TETs), including thymomas and thymic carcinomas (TC), are the most common primary tumors arising in the anterior mediastinum of the thymus (1). TETs are categorized into thymomas or thymic carcinomas, with thymomas further subdivided into types A, AB, B1, B2, and B3 (2). This classification, which ranks the malignancy potential from low to high, is crucial for guiding comprehensive patient care (3). Precise preoperative risk classification of TETs is vital for determining the appropriate treatment strategies and making accurate prognostic assessments (4). A simplified risk categorization, including low-risk thymoma (LRT) encompassing types A, AB, and B1 thymoma, high-risk thymoma (HRT) including types B2 and B3 thymoma, and TC, has been widely adopted in clinical practice to facilitate optimal therapeutic decision-making and management (5–7).

Contrast-enhanced chest computed tomography (CT) is an essential tool for preoperative evaluation of thymic tumors. As the most common and noninvasive imaging modality, it offers significant value in assessing tumor size, contours, and invasive characteristics (8, 9). However, the accuracy of thymic tumor evaluation can be influenced by the varying levels of experience among radiologists (10). Given the complexity and heterogeneity of TETs, differentiating their risk categories based solely on radiological findings can be challenging.

The application of radiomic features in machine learning models based on medical images has demonstrated promising diagnostic performance for thymic tumors. A previous study reported a promising area under the receiver operating characteristic (ROC) curve (AUC) of 0.744 for predicting high-risk TETs using a machine-learning approach based on radiomic and deep learning (DL) features extracted from ^18^F-FDG-PET images (11). Another study developed a combined nomogram incorporating clinical, radiomic, and DL features based on contrast-enhanced CT images to effectively differentiate between thymomas and thymic cysts, achieving a significantly high AUC value of 0.945 (12).

In recent years, computer-aided diagnostic models, particularly those utilizing DL, have gained considerable attention in medical imaging (13, 14). DL models, when trained on extensive medical imaging datasets, have shown remarkable progress in detecting abnormalities, diagnosing and classifying diseases, and predicting outcomes. Compared to conventional radiological methods, DL can automatically extract and analyze features such as tumor size, shape, texture, and their relationship with neighboring structures, providing a more comprehensive and objective assessment. DL has been successfully employed in clinical settings for the detection and analysis of lung nodules (15), fractures (16), brain tumors (17, 18), strokes (19), and coronary artery lesions (20), among others. Thus, DL has the potential to assist radiologists in overcoming subjectivity and variability in expertise, enabling more precise and consistent evaluation of thymic tumors.

We hypothesized that DL models, trained on multicenter venous-phase CT images of patients with TETs, could effectively differentiate risk categories according to the WHO classification system and enhance radiologists’ diagnostic performance. This study aimed to develop and validate an optimized DL model using data from multiple centers, evaluate its performance, and compare the diagnostic accuracy of radiologists with and without the aid of the DL model.

## Materials and methods

### Patient cohort

This study was approved by the Institutional Review Board (IRB) of the First Hospital of Shanxi Medical University (approval no. KYLL-2024-355) and the IRB of the Shanxi Cancer Hospital (approval no.202230). All data collection and analyses were conducted in accordance with the tenets of the Declaration of Helsinki. Since this is a retrospective study, the requirement for informed consent was waived by the Institutional Review Board (IRB) of the First Hospital of Shanxi Medical University and the IRB of the Shanxi Cancer Hospital.

**Figure 1:**
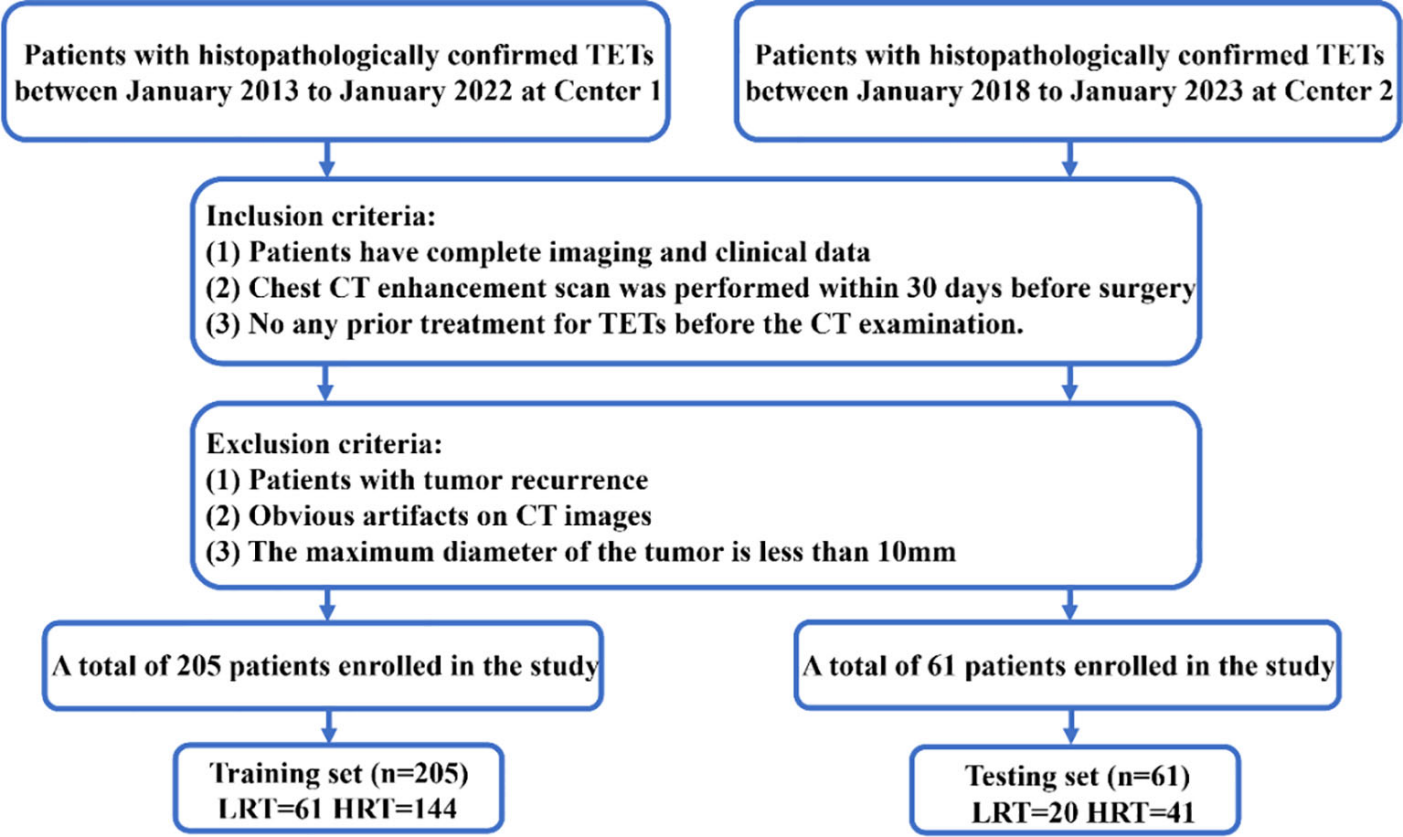
Flowchart of patient recruitment. LRT; HRT; Center 1, Shanxi Cancer Hospital; Center 2, The First Hospital of Shanxi Medical University.

We conducted a retrospective study on 266 patients who had undergone surgical resection and were pathologically diagnosed with TETs from two different centers (**Supplementary Table S1**), all of whom underwent contrast-enhanced chest CT (**Figure 1**).

The inclusion criteria for the patient cohort were as follows (1): Patients diagnosed with TETs and possessing complete imaging and clinical data;(2) Underwent a contrast-enhanced chest CT within 30 days before surgery.;(3) Had not undergone any previous treatment for TETs before the CT examination. The exclusion criteria were as follows:(1) Tumor recurrence;(2) Significant artifacts on CT images; (3) Maximum tumor diameter < 10 mm.

### Basic clinical and radiological characteristics

Data collection was standardized across both centers to ensure consistency. The following data were collected from each patient: (1)Clinical data: age gender, and relevant family medical history; (2)Radiological data: CT images obtained during the venous phase of contrast enhancement, are recognized as the optimal phase for evaluating mediastinal tumors. Images were collected in a digital format with consistent resolution and slice thickness; (3)Pathological data: Surgical pathology reports and biopsy results were recorded.

### CT image acquisition

Each patient underwent a multiphase contrast-enhanced chest CT scan in the supine position, following a deep breath using a multi-slice spiral CT apparatus. Comprehensive information regarding the imaging protocols employed at the two centers is provided in **Supplementary Table S2**.

Venous-phase images were selected because of their ability to delineate boundaries for the majority of thymic tumors, thereby enhancing lesion visualization and facilitating accurate segmentation of anterior mediastinal tumors (12, 21, 22).

### Radiomic model construction and validation

For comparison, venous-phase radiomics features of TETs were utilized to construct a risk categorization model using standard machine learning algorithms such as Logistic Regression, Support Vector Machine, K-Nearest Neighbor, Extra Trees, and Multilayer Perceptron. The final optimized machine-learning model was employed for further investigation.

**Figure 2:**
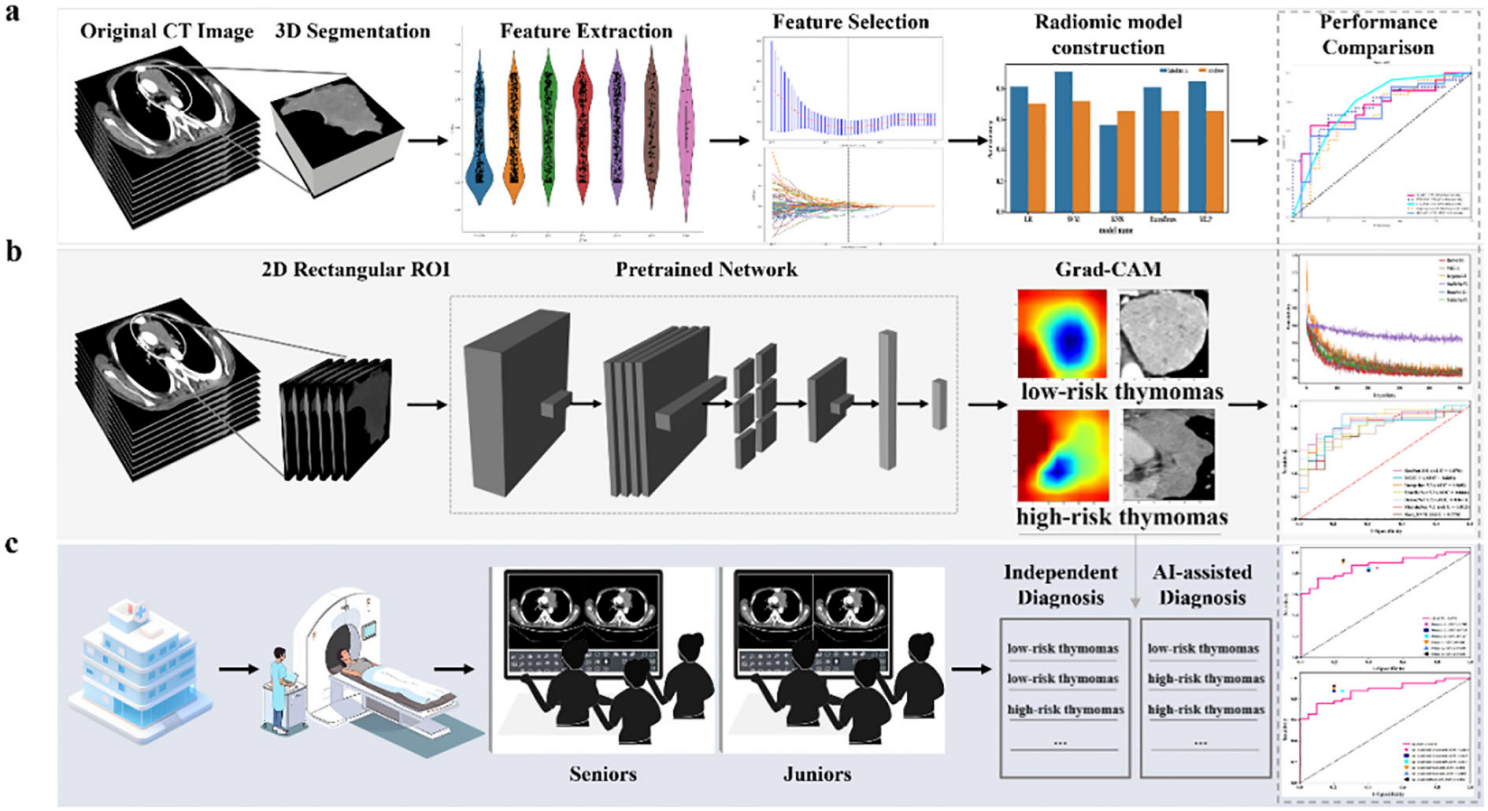
The overall pipeline of this study includes **(a)** constructing the baseline traditional machine learning model, **(b)** constructing the DL model using 2D-based venous-phase CT images as inputs to various pre-trained models (MobileNet V2, ShuffleNet V2, Inception V3, DenseNet 121, ResNet 101, and VGG 11). The model’s output predicted probabilities of LRT and HRT. In addition, gradient-weighted class activation mapping (Grad-CAM) was used for visualizing the decision-making process of the model; and **(c)** radiologists with different levels of experience performed comprehensive interpretations of LRT and HRT both with and without model assistance.

Two thoracic radiologists (Reader 1 and Reader 2, with five and eight years of experience in chest imaging, respectively) conducted image segmentation and were blinded to all clinical information. **Figure 2** summarizes the overall pipeline of this study. Reader 1 independently performed manual segmentation of all tumor regions of interest (ROIs) on each axial slice using open-source ITK-SNAP software (version 3.8.0, www.itksnap.org). To assess inter-rater reliability in the tumor segmentation process, Reader 1 and Reader 2 re-outlined the tumor ROIs in 30 randomly selected patients two weeks later. Intra-class correlation coefficients were also calculated.

The images underwent preprocessing to enhance feature reproducibility and eliminate the variance arising from different acquisition parameters. All images were resampled to a standardized voxel size of 1×1×1 mm using the B-spline interpolation algorithm. Voxel intensity values were discretized with a fixed bin width of 32 units, and the window width and level were set to 400 HU and 40 HU, respectively. Subsequently, radiomic features of the tumors were extracted from the Three-dimensional (3D) volumes of interest using the pyradiomics software package (version 3.1.0) on the Python platform (version 3.7).

After normalizing all radiomic features using z-standardization to achieve a normal distribution, a rigorous three-step process was implemented for feature selection.(1) The intraclass correlation coefficients were used to assess inter- and intra-observer reliabilities. Only radiomic features with coefficients greater than 0.85 were selected for further analysis.(2) Pearson’s correlation coefficients were calculated to quantify the relationships among highly redundant features. Features with an absolute correlation coefficient greater than 0.9 between any two were reduced to retain only one, eliminating redundancy.(3) The least absolute shrinkage and selection operator method with 5-fold cross-validation was used to identify features with nonzero coefficients. The regularization parameter (λ) was optimized based on criteria to ensure the selection of the most discriminative features.

Furthermore, to achieve a balanced representation of minority samples in the training set, the synthetic minority oversampling technique algorithm was utilized with the aim of a 1:1 ratio.

Finally, a machine learning model based on the SVM algorithm with a radial basis kernel was constructed for a risk categorization of TETs using the selected features. To comprehensively evaluate the predictive performance of the model, various metrics were employed: sensitivity, specificity, accuracy, NPV, PPV, and AUC.

### Development and visualization of DL models

In this study, six pre-trained DL architectures were utilized: DenseNet 121, ResNet 101, Inception V3, VGG 11, MobileNet V2, and ShuffleNet V2. These models were chosen because of their diverse architectures and proven effectiveness in various medical image analysis tasks.

Two-dimensional (2D) rectangular ROIs encompassing the maximum extent of the tumor were cropped slice-by-slice from raw CT images, guided by the 3D segmentation mask of the tumor.

In the training set, 410 images were derived from 205 samples (Center 1). Each image represented a 2D rectangular ROI cropped from the layer with the maximum tumor area and its adjacent layer at a fixed interval of +1 mm. This approach not only maximized the utility of limited data by enhancing training diversity but also preserved spatial tumor continuity, thereby effectively capturing key heterogeneity near the diagnostically critical regions. Potential selection bias was minimized by selecting the primary slice based on the objective and measurable criterion of maximum tumor area, systematically choosing the adjacent slice (+1 mm), and adhering to a predefined radiologist annotation protocol. The external testing set included 61 single-slice images (largest tumor area) from 61 samples (Center 2). All images were preprocessed with a 400 HU window width and 40 HU window level to ensure feature consistency. Image cropping and preprocessing were performed on the OnekeyAI platform (v3.2.6), in accordance with prior studies (23, 24). After preprocessing, z-score normalization was applied to all 2D images. Subsequently, all images were resized to 299 × 299 pixels for Inception V3 and 224 × 224 pixels for the other CNN models. During the training phase, data-augmentation techniques were utilized to enhance the diversity of the training dataset and mitigate the risk of overfitting. This includes applying various transformations to the original images, such as rotation, translation, and scaling, thereby effectively increasing the number of training samples.The detailed image processing procedure can be found in the **Supplementary Appendix S1**.

To optimize model performance, we employed a grid search method to systematically explore the hyperparameter space. The training procedure utilized an initial learning rate of 0.01, spanning 500 epochs with 13,000 iteration steps and a batch size of 16. To enhance the interpretability of the DL models, we applied Grad-CAM. This technique visualizes the decision-making process of these models by highlighting the regions in the input image that most influence the final decision. Viewing the Grad-CAM results aids in understanding how the models distinguish between LRT and HRT. The detailed 2D Deep Learning Model Training can be found in the **Supplementary Appendix S1**.

### Conventional visual diagnosis of radiologists and DL model aiding diagnosis

We employed a dual-track evaluation approach that combined the visual assessments of experienced radiologists with the assistance of a DL model. We have successfully recruited and assembled a team of six thoracic imaging radiologists, which has been divided into two groups: junior and senior groups (<3 years and >10 years of experience, respectively), each consisting of three radiologists. Both groups of radiologists, blinded to the histopathological results underwent a brief training session to familiarize themselves with the specific tasks and protocols of the study. Initially, the two groups of radiologists independently assessed low-risk or high-risk thymomas based on CT imaging findings, which encompass various aspects, such as size, shape, intratumoral density, contour, enhancement pattern, tissue invasion, and other relevant factors (25). Subsequently, after a month, the radiologists in each group conducted a reassessment of low-risk or high-risk thymomas utilizing the diagnoses and heatmaps generated by the optimal DL model developed in this study. A comparison was conducted between the diagnosis provided by the radiologist and that generated by the model. In cases of discrepancy, the radiologist had the option to either accept the model’s diagnosis or maintain their own as the definitive diagnosis. Additionally, a comparative analysis was performed on each radiologist’s two distinct evaluations.

### Statistical analysis

Continuous variables were summarized using mean ± standard deviation. The assumption of normal distribution was examined using the Shapiro-Wilk test. If not violated normality, comparisons between groups were conducted using the nonparametric Mann–Whitney U test. Categorical variables were presented as frequencies and percentages, and comparisons were made using the chi-square test. The DeLong method was used to compare the ROC curves among different models. Statistical significance was set at a two-tailed *p*-value <0.05. Statistical analyses were conducted using SPSS (version 25.0, IBM) and Python (version 3.7).

## Results

### Clinical and conventional CT characteristics of patients

In this multicenter study, we performed a comprehensive analysis of baseline clinical data and conventional CT characteristics of 266 patients with TETs. As shown in **Table 1**, no statistically significant differences in age, sex, tumor size, volume, calcification, or degree of enhancement were observed between the LRT and HRT groups in the training and testing sets. However, a significant difference in the margin and enhancement patterns was observed between the two groups in both datasets.

**Table 1:** Baseline clinical-radiological features of the datasets.

Characteristics	Training set			Testing set		
	Low-risk thymomas (n=61)	High-risk thymomas (n=144)	*p*	Low-risk thymomas (n=20)	High-risk thymomas (n=41)	*p*
Age (years)						
Mean±SD	56.85±9.702	54.89±11.085	0.231	56.00±13.412	52.93±12.863	0.391
Gender						
Male	27 (44%)	85 (59%)	0.052	9 (45%))	29 (71%)	0.051
Female	34 (56%)	59 (41%)		11 (55%)	12 (29%)	
Maximal diameter (cm)						
Mean±SD	7.780±2.919	8.096±2.797	0.490	5.4540±3.62337	5.2854±2.21332	0.850
Minimal diameter (cm)						
Mean±SD	4.272±1.800	4.264±1.502	0.972	2.8815±2.01511	2.6954±1.13150	0.704
Tumor volume (cm3)						
Mean±SD	194.764±269.033	151.149±145.462	0.236	97.46325±169.779332	44.48810±48.976316	0.186
Contour						
Smooth	21 ( (34%)	50 (35%)	0.898	8 (40%)	18 (44%)	0.266
Lobulated	28 (46%)	70 (49%)		8 (40%)	14 (34%)	
Irregular	12 (20%)	24 (16%)		4 (20%)	9 (22%)	
Margin						
Clear	52 (85%)	44 (31%)	0.000	19 (95%)	15 (37%)	0.000
Unclear	9 (15%)	100 (69%)		1 (5%)	26 (63%)	
Calcification						
No	52 (85%)	105 (73%)	0.057	19 (95%)	32 (78%)	0.093
Yes	9 (15%)	39 (27%)		1 (5%)	9 (22%)	
Enhancement pattern						
Heterogeneous	36 (59%)	106 (74%)	0.038	7 (35%)	15 (37)	0.015
Homogeneous	25 (41%)	38 (26%)		13 (65%)	26 (63%)	
Enhancement degree						
Minimal	16 (26%)	63 (44%)	0.058	15 (75%)	33 (80%)	0.690
Moderate	25 (41%)	46 (32%)		4 (20%)	6 (15%)	
Severe	20 (33%)	35 (24%)		1 (5%)	2 (5%)	

### Performance evaluation of a machine learning model based on radiomics features

Radiomic features were extracted from each segmentation and categorized into shape, first-order, and texture features. A total of 1,743 handcrafted features were obtained, comprising 14 shape features, 342 first-order features, and 1,387 texture features (**Supplementary Figures S1a, b**). Following feature selection, 20 radiomics features–comprising 2 shape features, 5 first-order features, and 13 texture features were retained for the development of the machine learning model. The coefficients and mean standard errors from the fivefold validation are presented in **Supplementary Figure S2a** and **Supplementary Figure S2b**, respectively. The feature coefficients are shown in **Supplementary Figure S2c**.

The results obtained from various machine learning algorithms are presented in **Supplementary Table S3**, and the corresponding ROC curves are illustrated in **Supplementary Figure S3**. Based on these results, the SVM machine learning model exhibited superior performance in accurately distinguishing between LRT and HRT on both the training and testing datasets. In the training set, the SVM model achieved an AUC of 0.928 (95% CI: 0.879-0.977), accuracy of 0.912, sensitivity of 0.910, specificity of 0.918, PPV of 0.963, NPV of 0.812, and F1 Score of 0.936. In the external testing set, the model achieved an AUC of 0.776 (95% CI: 0.6576-0.8936), accuracy of 0.721, sensitivity of 0.683, specificity of 0.800, PPV of 0.875, NPV of 0.552, and F1 Score of 0.767. set.

### Evaluation of DL model performance and radiologists’ diagnostic performance with DL

**Figure 3:**
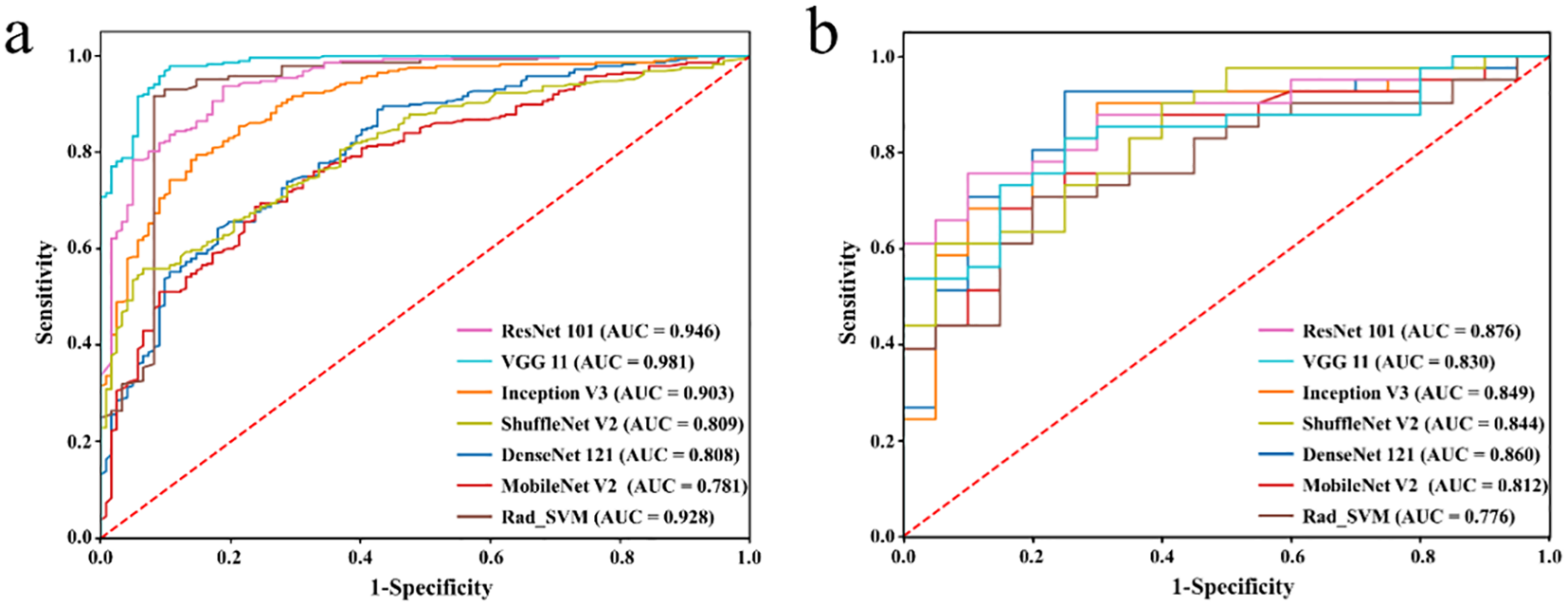
Receiver operating characteristic curves of different DL models in the training set **(a)** and testing set **(b)**, respectively.

**Figure 4:**
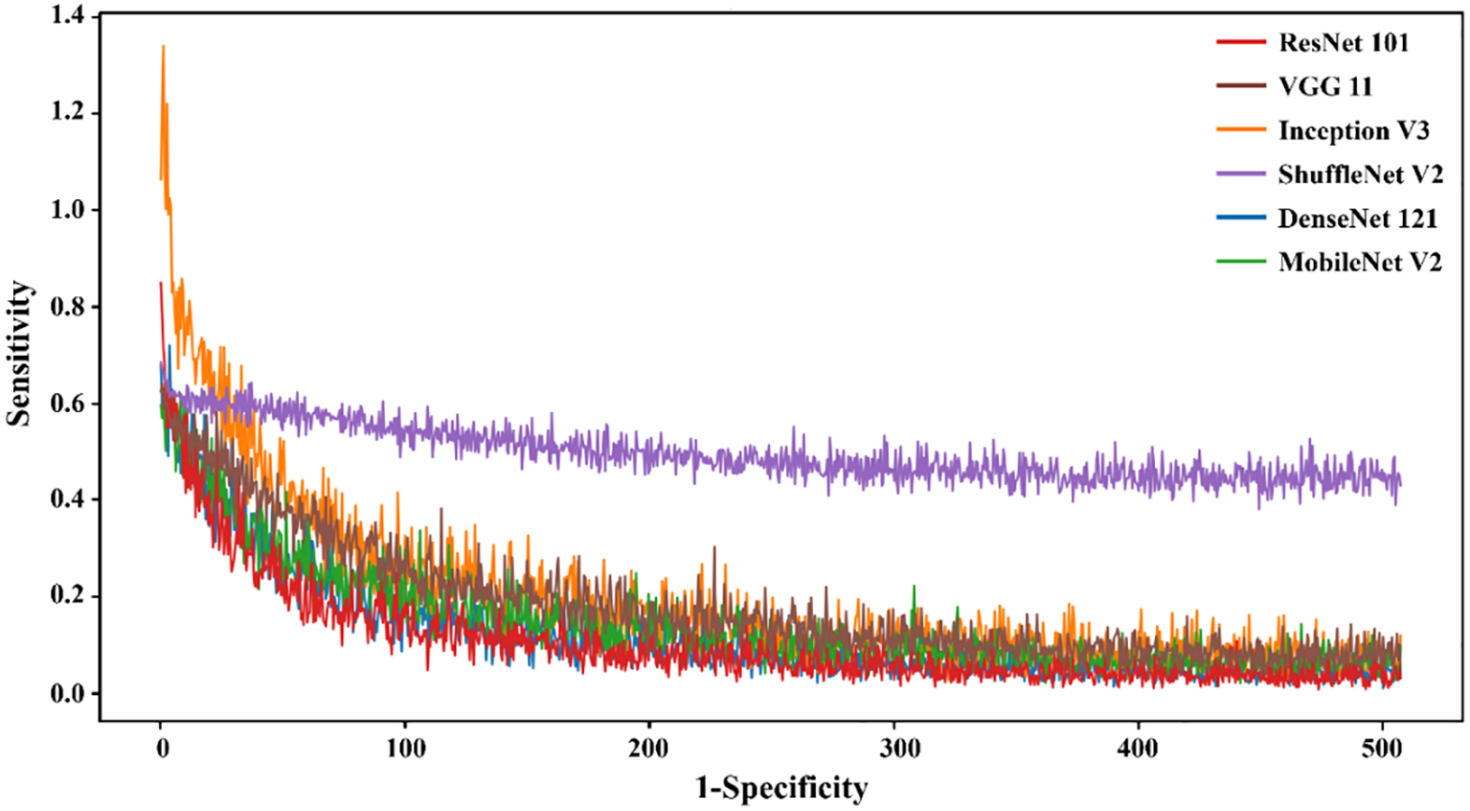
The loss value of different DL models in the training set varied with the iteration steps.

The performance of deep learning models was summarized in **Table 2**, while the corresponding ROC curves and Loss curves were presented in **Figures 3** and **4**, respectively.

**Table 2:** The performance comparison of different models.

Model	Set	AUC (95% CI)	Accuracy	Sensitivity	Specificity	PPV	NPV
ResNet 101	Training	0.946 [0.9231 - 0.9682]	0.888	0.948	0.746	0.898	0.858
	Testing	0.876 [0.7908 - 0.9604]	0.820	0.878	0.700	0.857	0.737
VGG 11	Training	0.981 [0.9704 - 0.9922]	0.946	0.965	0.902	0.959	0.917
	Testing	0.830 [0.7279 - 0.9331]	0.803	0.854	0.700	0.854	0.700
Inception V3	Training	0.903 [0.8717 - 0.9337]	0.849	0.910	0.705	0.879	0.768
	Testing	0.849 [0.7449 - 0.9526]	0.820	0.902	0.650	0.841	0.765
ShuffleNet V2	Training	0.809 [0.7676 - 0.8512]	0.759	0.910	0.402	0.782	0.653
	Testing	0.844 [0.7442 - 0.9436]	0.820	0.976	0.500	0.800	0.909
DenseNet 121	Training	0.808 [0.7626 - 0.8526]	0.788	0.896	0.533	0.819	0.684
	Testing	0.860 [0.7570 - 0.9625]	0.803	0.829	0.750	0.872	0.682
MobileNet V2	Training	0.781 [0.7345 - 0.8279]	0.744	0.861	0.467	0.792	0.588
	Testing	0.812 [0.7013 - 0.9218]	0.770	0.829	0.650	0.829	0.650
Rad_SVM	Training	0.928 [0.8786 - 0.9773]	0.898	0.958	0.754	0.902	0.885
	Testing	0.776 [0.6576 - 0.8936]	0.738	0.878	0.450	0.766	0.643

PPV, positive predictive value; NPV, negative predictive value; SVM, support vector machine.

**Figure 5:**
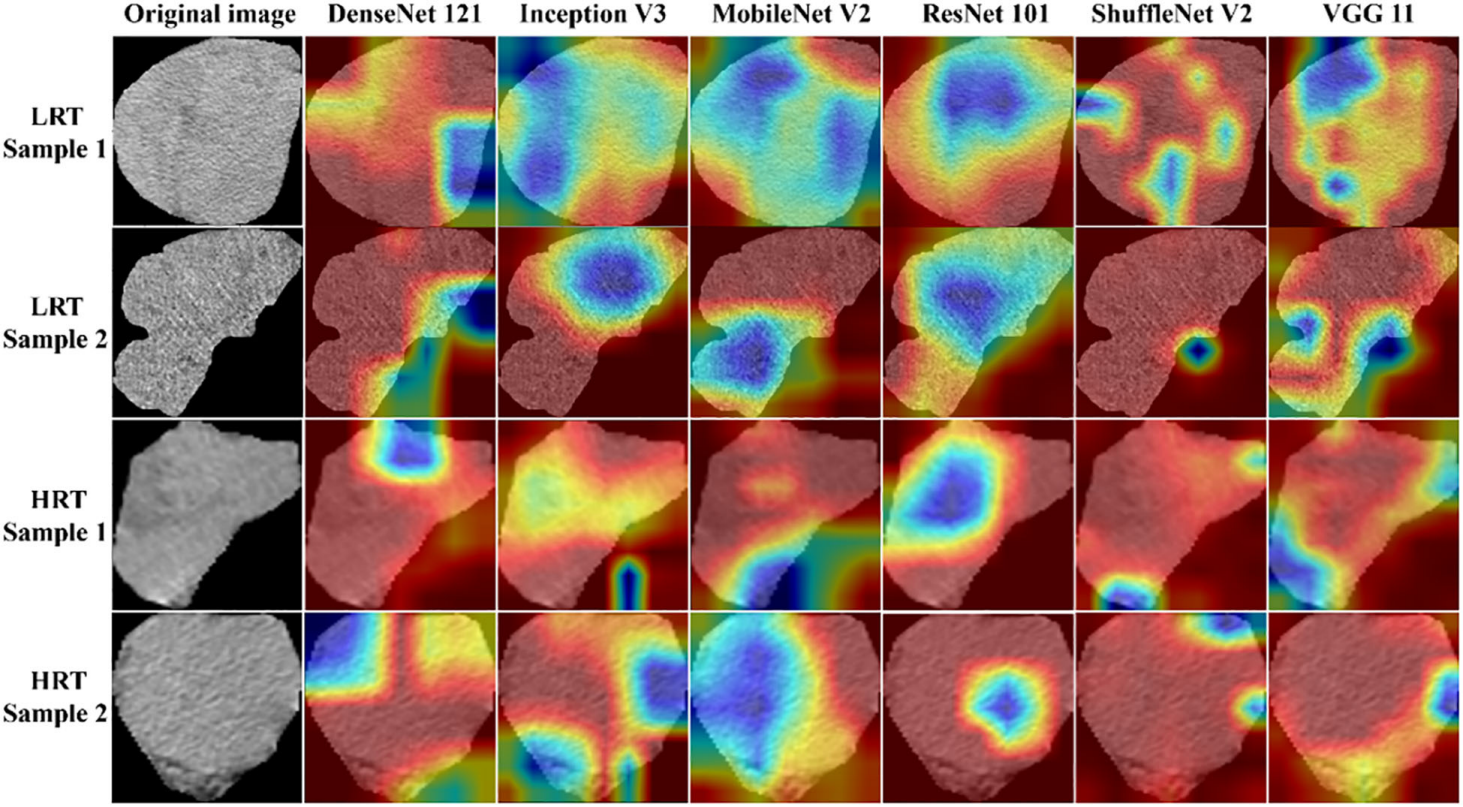
The attention regions of different DL models in CT image analysis for low-risk and high-risk thymomas; LRT, low-risk thymoma; HRT, high-risk thymoma.

The results demonstrated that the ResNet 101 model exhibited relatively better comprehensive performance compared to other CNN models in the external testing set. Specifically, it achieved the highest classification performance with an AUC of 0.876 (95% CI: 0.791-0.960), accuracy of 0.820, sensitivity of 0.878, specificity of 0.700, PPV of 0.857, and NPV of 0.737. Furthermore, in the external testing set, the ResNet 101 model consistently outperformed the SVM model based on radiomics features in terms of AUC, accuracy, specificity, PPV, and NPV. However, both models exhibited equal sensitivity. The loss curves demonstrated that the ResNet 101 model exhibited minimal loss values, indicating a reduced occurrence of errors during training and faster convergence compared to all other CNN models investigated in this study. Additionally, Grad-CAM analysis (as shown in **Figure 5**) revealed that the ResNet 101 model exhibited more distinct attention regions and primarily focused on both the boundary and internal areas of the tumor compared to other CNN models.

**Figure 6:**
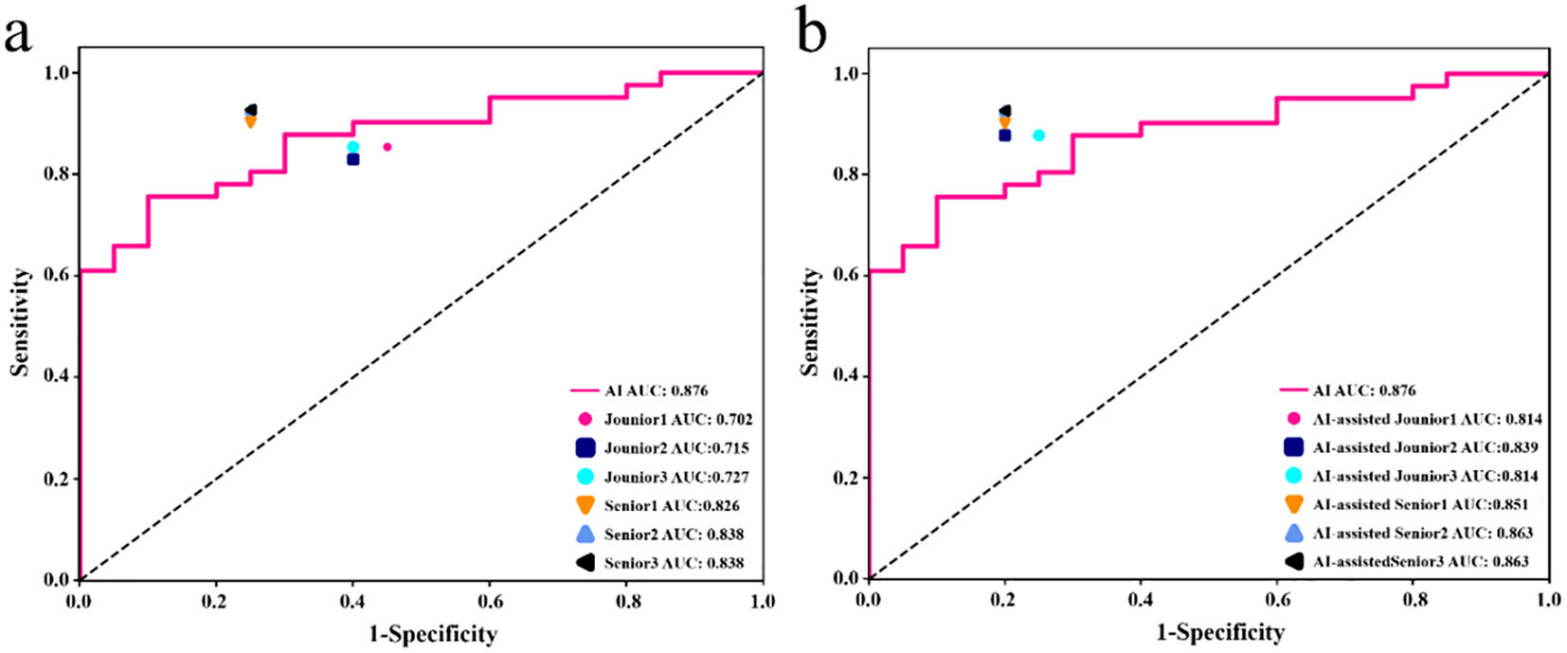
In the testing set, the performance of junior and senior radiologists in differentiating LRT from HRT was compared: **(a)** analysis conducted without AI assistance, and **(b)** analysis conducted with the support of an AI (ResNet 101 model).

The diagnostic performance of junior and senior radiologists in distinguishing between LRT and HRT, with or without assistance from the ResNet 101 model, is summarized in **Table 3**. The results demonstrated a significant improvement in the overall performance of junior radiologists when assisted by the model. As shown in **Figure 6**, junior radiologists initially had poorer diagnostic performance than their senior counterparts. However, with AI assistance, junior radiologists exhibited a significant improvement in diagnostic performance compared to those without AI assistance, as determined by DeLong’s test (**Supplementary Table S4**). Although the AUC values for senior radiologists improved with AI assistance, no significant differences were found according to DeLong’s test. Additionally, there was no statistical difference in diagnostic performance between AI-assisted junior and senior radiologists. These results indicate that the diagnostic performance of AI-assisted junior radiologists can significantly narrow the gap with their senior counterparts.

**Table 3:** Performance comparison between AI and radiologists and between radiologists with and without AI assistance.

Group	Set	AUC (95% CI)	Accuracy	Sensitivity	Specificity	PPV	NPV
AI	Testing	0.876 [0.7908 - 0.9604]	0.820	0.878	0.700	0.857	0.737
Jounior1	Testing	0.702 [0.5773 - 0.8264]	0.754	0.854	0.550	0.795	0.647
Jounior2	Testing	0.715 [0.5900 - 0.8393]	0.754	0.829	0.600	0.810	0.632
Jounior3	Testing	0.727 [0.6038 - 0.8498]	0.770	0.854	0.600	0.814	0.667
Senior1	Testing	0.826 [0.7186 - 0.9339]	0.852	0.902	0.750	0.881	0.789
Senior2	Testing	0.838 [0.7330 - 0.9438]	0.869	0.927	0.750	0.884	0.833
Senior3	Testing	0.838 [0.7330 - 0.9438]	0.869	0.927	0.750	0.884	0.833
AI-assisted Jounior1	Testing	0.814 [0.7043 - 0.9238]	0.836	0.878	0.750	0.878	0.750
AI-assisted Jounior2	Testing	0.839 [0.7358 - 0.9423]	0.852	0.878	0.800	0.900	0.762
AI-assisted Jounior3	Testing	0.814 [0.7043 - 0.9238]	0.836	0.878	0.750	0.878	0.750
AI-assisted Senior1	Testing	0.851 [0.7502 - 0.9522]	0.869	0.902	0.800	0.902	0.800
AI-assisted Senior2	Testing	0.863 [0.7648 - 0.9620]	0.885	0.927	0.800	0.905	0.842
AI-assisted Senior3	Testing	0.863 [0.7648 - 0.9620]	0.885	0.927	0.800	0.905	0.842

PPV, positive predictive value; NPV, negative predictive value.

## Discussion

The retrospective multicenter study presented here demonstrated that a CNN model based on contrast-enhanced CT, specifically ResNet 101, achieved high diagnostic performance with an AUC of 0.876. This model notably surpassed traditional radiomics methods in accurately distinguishing between LRT and HRT. Additionally, the ResNet 101 model proved valuable in improving diagnostic performance among radiologists, thereby bridging the gap between junior and senior radiologists’ diagnostic outcomes.

The precise preoperative risk classification of TETs is of critical importance for guiding treatment strategies and prognostic evaluations, as histopathological subtypes significantly impact clinical decision-making (5). Traditional non-invasive imaging modalities such as CT and MRI heavily depend on radiologists’ subjective interpretations; however, the inherent heterogeneity and complex morphology of TETs often pose substantial challenges to accurate risk stratification. This challenge is further exacerbated by interobserver variability stemming from differing levels of diagnostic expertise. Although fine-needle aspiration (FNA) serves as a diagnostic gold standard, its invasive nature and associated procedural risks limit its widespread clinical application. The present multicenter study illustrates that a contrast-enhanced CT-based DL framework achieves excellent diagnostic performance in distinguishing LRT and HRT, thus providing a promising non-invasive alternative.

DL models have emerged as powerful tools in the field of medical image analysis, offering several advantages for tumor risk classification (26). DL models automated the extraction and analysis of extensive imaging datasets, overcoming the limitations of human observers in visual perception and experience (27–30). This enables the capability to facilitate the detection of subtle patterns and features that may be imperceptible to radiologists, thus improving diagnostic performance (27). For TETs, the application of CT-based DL models has the potential to significantly improve the accuracy and consistency of preoperative risk categorization. Several studies have convincingly demonstrated the effectiveness of CT-based DL radiomics models in differentiating the histologic subtypes of thymic tumors preoperatively (5, 12, 31). The study by Ye Sung Moon et al. utilized 3D U-Net++ for automatic segmentation of thymoma based on CT images, demonstrating the applicability of CNN DL techniques in TET risk classification (32). They achieved an optimal AUC of 0.81 was achieved using 3D ResNet50, outperforming the other four classical CNNs (3D SE ResNext50, 3D DenseNet121, and 3D VGG19) in TETs risk classification (32).

The present study used six pre-trained CNN models (ResNet 101, DenseNet 121, Inception V3, ShuffleNet V2, VGG11, and MobileNet V2) with transfer learning to differentiate between LRT and HRT based on the WHO classification. Ultimately, the ResNet 101 model demonstrated superior diagnostic performance and robust generalization compared to other classical CNNs. These varied outcomes arise from the heterogeneous internal architecture of CNN models (33). ResNet 101 effectively handles deep network architectures by incorporating residual connections, which enable the model to learn identity mappings and train deeper networks while mitigating the issue of vanishing gradients. Furthermore, in comparison to traditional radiomics models based on SVM, the ResNet 101 model also demonstrated better diagnostic performance because of its ability to capture intricate relationships that might be overlooked by radiomics models. Grad-CAM is a visualization technique that aids clinicians in better understanding the decision-making process of DL models and identifying potential errors or biases by highlighting important regions in the input images. Grad-CAM visualization in this study revealed that the ResNet 101 model primarily focused on the internal regions and boundaries of the tumor when distinguishing between LRT and HRT. The focused regions are pivotal for radiologists to accurately observe and assess the characteristics and extent of tumors.

Although this study revealed that DL models in medical image analysis, especially risk classification of TETs, their role should be seen as complementary rather than as a replacement for radiologist expertise. Despite achieving high diagnostic accuracy, DL models still exhibit errors or biases that need to be addressed. However, DL has the potential to democratize radiology by providing less experienced radiologists in under-resourced areas access to specialized knowledge and expertise (34). The findings of this study demonstrate a significant enhancement in the diagnostic performance of junior radiologists aided by a DL model for risk classification in the external testing set compared to senior radiologists. Furthermore, the diagnostic results achieved by juniors radiologists approached that of their senior counterparts. Therefore, DL models have the potential to improve the diagnostic performance of radiologists in preoperative risk classification of TETs and bridge the gap between junior and senior radiologists.

Despite the promising findings of this study, several limitations and opportunities for improvement need to be addressed. First, the retrospective nature of our study limits the generalizability of our findings. To overcome this limitation, we plan to conduct prospective multicenter studies to validate our models in a more diverse patient population. Second, we aimed to expand our analysis by further subtyping TETs and incorporating additional clinical information, such as lymph node and organ metastasis. This will enrich our DL models and enhance their accuracy and robustness. Furthermore, we plan to integrate multiradiomics and multimodal imaging (including US, CT, and MR imaging) to further improve the precision and personalization of patient care in the medical field.

In conclusion, the development of a contrast-enhance CT-based DL model shows significant potential for improving the accuracy and consistency of preoperative risk classification for TETs. This model effectively overcomes the limitations of traditional radiological evaluations, reduces reliance on radiologist expertise, and enables more precise therapeutic decision-making. The multicenter study design further validates the robustness and generalizability of this approach, providing a valuable tool for managing TETs in clinical practice.

## Data Availability

The raw data supporting the conclusions of this article will be made available by the authors, without undue reservation.
