# Supplementary material for: Contrast-enhanced CT-based deep learning model assists in preoperative risk classification of thymic epithelial tumors
Source: Front Oncol. 2025 Jul 31;15:1616816. doi: 10.3389/fonc.2025.1616816 (PMC12350125; doi:10.3389/fonc.2025.1616816)
Supplement: Supplementary file 1 [file DataSheet1.docx]

Supplementary

**Appendix S1**

**Image Cropping and Preprocessing Workflow:**

Image standardization preprocessing was performed to minimize data variability across multi-center cohorts and prepare suitable inputs for the training of deep learning models and radiomics feature extraction.

Following manual segmentation in ITK-SNAP, the CT images from each sample were exported as Three-dimensional (3D)NIfTI files (.nii.gz). A systematic two-stage image normalization protocol was then applied: (a) Isotropic Resampling: All 3D NIfTI volumes were spatially normalized to a 1×1×1 mm³ isotropic voxel grid using cubic B-spline interpolation, ensuring spatial consistency across heterogeneous datasets; (b) Intensity Standardization: Voxel intensity values were quantized with a fixed bin width of 32 HU. Subsequently, a standardized mediastinal windowing protocol (window level = 40 HU, window width = 400 HU) was applied to preserve diagnostic interpretability.

Two-dimensional (2D) regions of interest (ROIs) containing the tumor were sequentially cropped layer-by-layer from 3D NIfTI files according to the 3D segmentation mask. This step ensured that the tumor region remained the focal point of the analysis.

**Resizing Methodology:**

Standardization: All 2D images were normalized using Z-score standardization to eliminate pixel intensity variations.

Resizing Strategy: Images were resized to 299×299 pixels for Inception V3 and to 224×224 pixels for other CNN models (e.g., ResNet 101, DenseNet 121, Inception v3, VGG 11, MobileNet v2 , and ShuffleNet v2).

Interpolation Algorithm: Bilinear interpolation was used for resizing due to its balance of computational efficiency and distortion control.

Aspect Ratio and Padding: Images were resized directly to target dimensions without preserving aspect ratios strictly. Edge padding (filling blank regions with edge pixel values) was applied to avoid non-medical artifacts.

Mitigation of Distortion and Deformation: The tumor was kept centered in resized images to minimize edge distortions. Data augmentation (e.g., random rotation, translation) during training improved the model's resilience to size variations.

**Appendix S2**

**2D Deep Learning Model Training Workflow:**

In this study, to ensure robust generalization across heterogeneous patient cohorts with inherent data variability, we adopted a transfer learning technique initialized using ImageNet-pretrained weights. This strategy enabled efficient feature extraction while mitigating overfitting risks.

2D images, after undergoing standardized preprocessing, represented the spatial dimensions of the cropped region of interest (ROI), which contained the tumor's maximal dimension. These images were subsequently processed by a suite of pre-trained convolutional neural network (CNN) architectures, including ResNet 101, DenseNet 121, Inception v3, VGG 11, MobileNet v2 , and ShuffleNet v2. Leveraging transfer learning, all models were initialized with weights pre-trained on the ImageNet dataset, a corpus renowned for its vast visual diversity and rich hierarchical feature representations, to enhance their generalization capability for medical imaging tasks. Training proceeded with a batch size of 16, optimized for the 12 GB memory capacity of the NVIDIA GeForce RTX 4070 GPU used.

**Hyperparameter Configuration of DL Model:**

Central to our methodology was a dynamic learning rate schedule employing cosine decay with warm restarts, mathematically formulated as:

$$\eta_{t}=\eta_{min}^{i}+\frac{1}{2}\left( \eta_{max}^{i}-\eta_{min}^{i} \right)\left( 1+\cos\left( \frac{T_{cur}}{T_{i}}\pi\right) \right)$$

Where and define the learning rate bounds, and Ti =30 represents the epoch interval for cyclic restarts. This schedule facilitates adaptive convergence by smoothly modulating the learning rate between extrema, promoting thorough exploration of parameter space.

Additional hyperparameters were meticulously configured:

Optimizer: Stochastic Gradient Descent (SGD) with momentum (0.9) to stabilize gradient updates.

Loss Function: Softmax cross-entropy to handle multi-class classification tasks.

Regularization: Integrated L2 weight decay (1×10−4 ) to prevent overfitting.


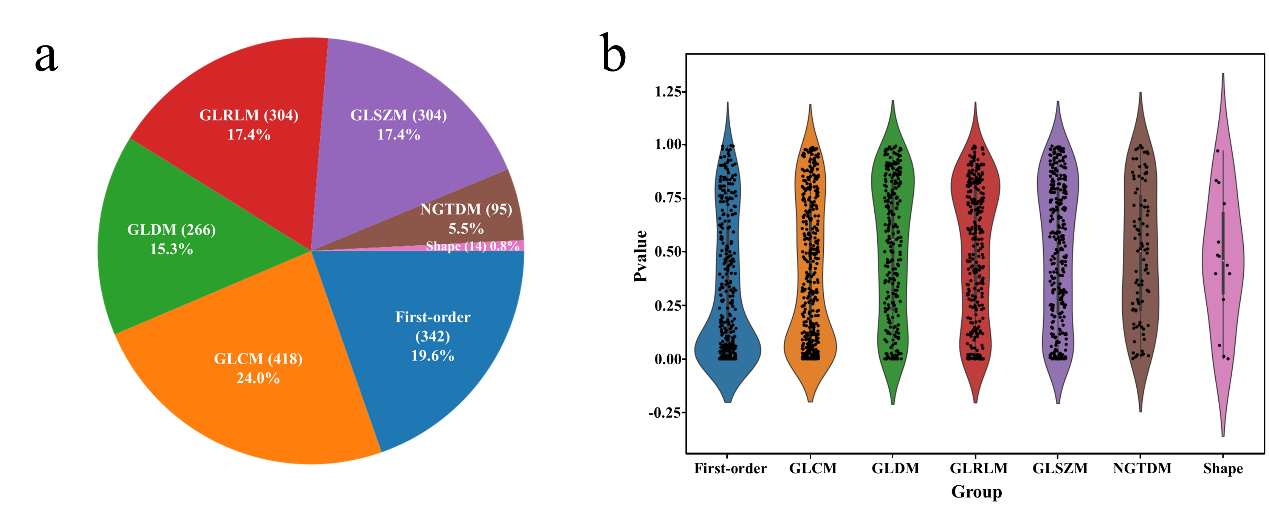


Fig. S1 (a) The 1743 radiomic features extracted included 14 shape features, 342 first-order features, and 1387 texture features (418 GLCM, 266 GLDM, 304 GLRLM, 304 GLSZM, and 95 NGTDM). (b) The distribution of all radiomics features' corresponding p values indicated that the first-order and texture (GLCM) features have strong discriminative power for risk categorization of TETs.


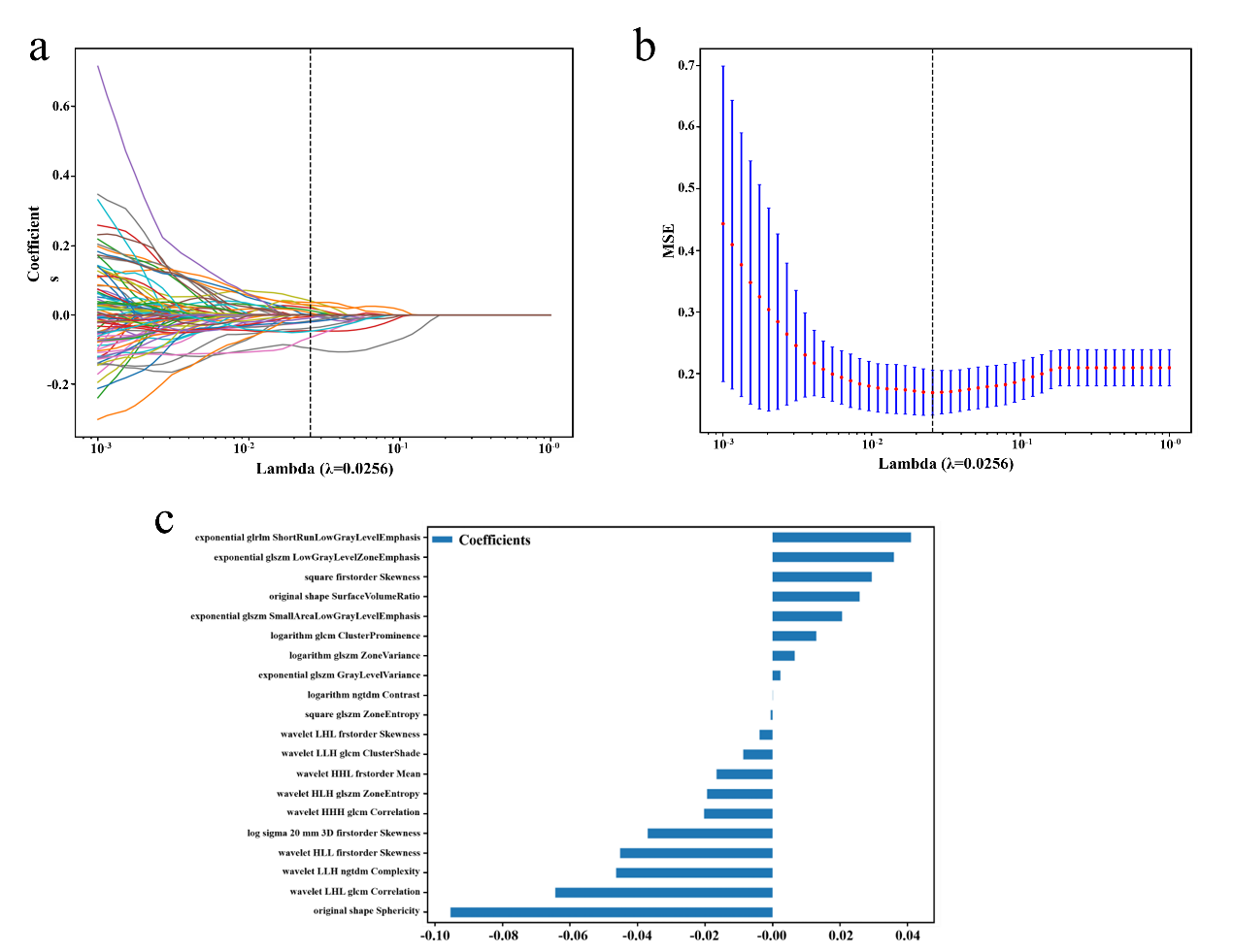


Fig. S2 The final features selected by the LASSO method with 5-fold cross-validation, including (a) coefficients from 5-fold cross validation, (b) MSE values from 5-fold cross validation, and (c) feature coefficients.


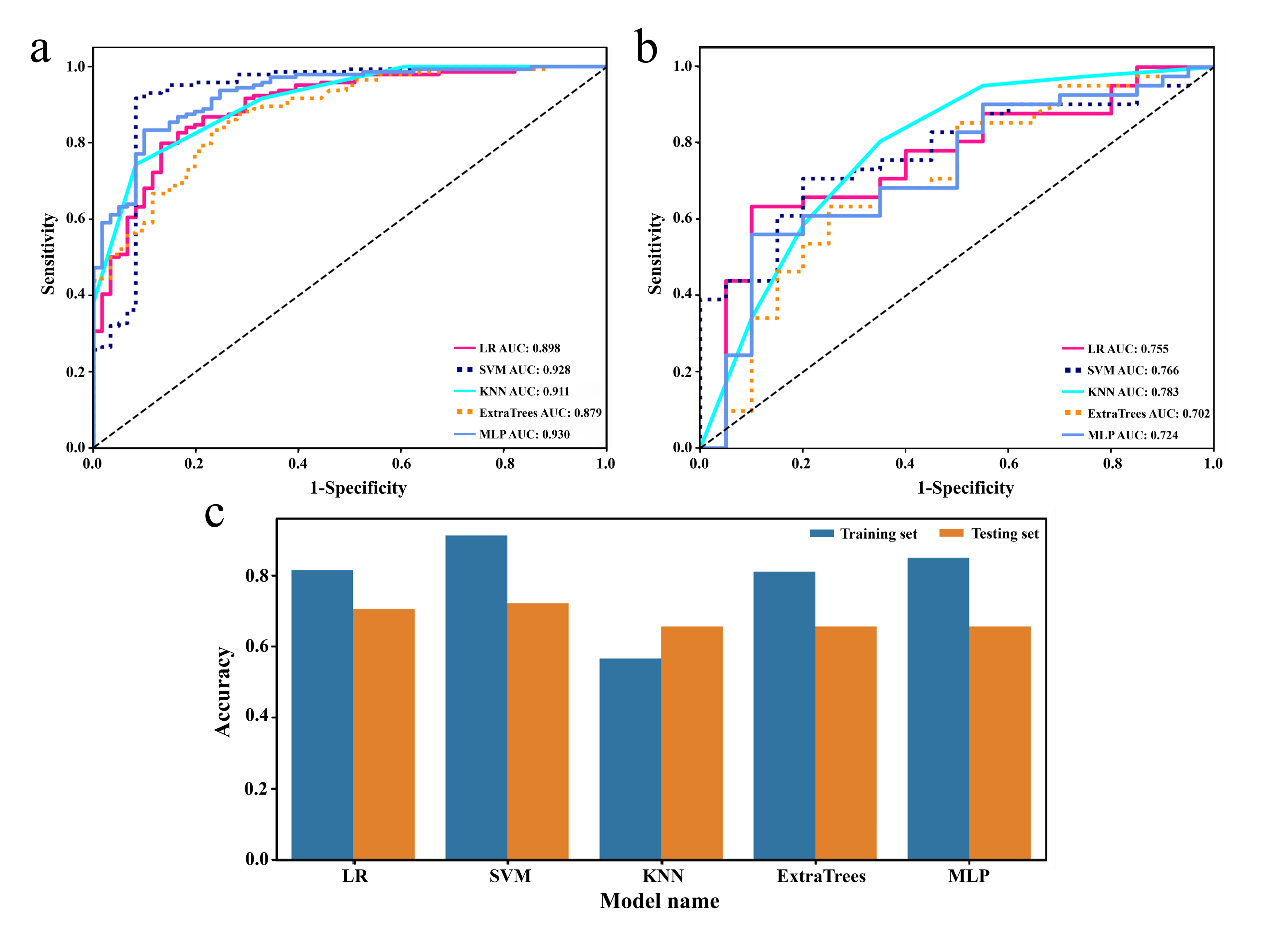


Fig. S3 ROC curves of different radiomic models in the training set (a) and testing set (b), respectively. (c) The accuracy of different radiomic models in the training and testing sets.

Table S1 Histopathological types and numbers of the two centers.

|  | Number | WHO pathological subtypes | | | | | | Traditional risk stratification | |
| --- | --- | --- | --- | --- | --- | --- | --- | --- | --- |
| Data set |  | Type | Type | Type | Type | Type | TC | LRT | HRT |
| (year) | (%) | A | AB | B1 | B2 | B3 |  | (A/AB/B1) | (B2/B3/TC) |
| All data | 266 | 26 | 31 | 25 | 61 | 43 | 80 | 82 | 184 |
| (2013-2023) | 100% | 9.8% | 11.7% | 9.4% | 22.9% | 16.2% | 30.1% | 30.8% | 69.2% |
| Center 1 | 205 | 19 | 26 | 17 | 40 | 28 | 75 | 62 | 143 |
| (2013-2022) | 77% | 9.3% | 12.7% | 8.3% | 19.5% | 13.7% | 36.6% | 30.2% | 69.8% |
| Center 2 | 61 | 7 | 5 | 8 | 21 | 15 | 5 | 20 | 41 |
| (2018-2023) | 23% | 11.5% | 8.2% | 13.1% | 34.4% | 24.6% | 8.2% | 32.8% | 67.2% |

Table S2 The CT equipment models and scan protocols of the two centers.

| Parameters | Center 1 | | | Center 2 | | |
| --- | --- | --- | --- | --- | --- | --- |
| CT scanners | GE Discovery CT750 HD | GE LightSpeed 16 | SOMATOM Force | SOMATOM Force | Iqon Spectral CT | GE Revolution 256 |
| Tube voltage | 120 kV | 120 kV | 100-120 kV | 100-120 kV | 120 kV | 120 kV |
| Tube current | Automatic  tube-current | Automatic  tube-current | Automatic  tube-current | Automatic  tube-current | Automatic  tube-current | Automatic  tube-current |
| Gantry rotation time | 0.6 s | 0.5 s | 0.28 s | 0.28 s | 0.33 s | 0.28 s |
| Detector collimation | 64×0.625 mm | 16×0.625 mm | 2×96×0.6 mm | 2×96×0.6 mm | 128×0.6 mm | 256×0.625 mm |
| Section thickness | 5 mm | 5 mm | 5 mm | 5 mm | 5 mm | 5 mm |
| Section interval | 5 mm | 5 mm | 5 mm | 5 mm | 5 mm | 5 mm |
| Image matrix | 512×512 | 512×512 | 512×512 | 512×512 | 512×512 | 512×512 |
| Contrast agent type | Ioversol | Ioversol | Ultravist | Ioversol | Ioversol | Ultravist |
| Contrast agent concentration | 350 mgI/mL | 350 mgI/mL | 370 mgI/mL | 350 mgI/mL | 350 mgI/mL | 370 mgI/mL |
| Contrast agent dosage | 1.2 mL/kg | 1.2 mL/kg | 1.0 mL/kg | 1.2 mL/kg | 1.2 mL/kg | 1.0 mL/kg |
| Contrast agent infused rate | 3.0 mL/s | 3.0 mL/s | 3.0 mL/s | 3.0 mL/s | 3.0 mL/s | 3.0 mL/s |
| Venous phase scan | 70s after the contrast injection | 70s after the contrast injection | 70s after the contrast injection | 70s after the contrast injection | 70s after the contrast injection | 70s after the contrast injection |

Table S3 The performance comparison of different machine learning models using radiomic features.

| Model |  | AUC (95%CI) | Accuracy | Sensitivity | Specificity | PPV | NPV |
| --- | --- | --- | --- | --- | --- | --- | --- |
| LR | Training | 0.898 ［0.8513 - 0.9438］ | 0.815 | 0.792 | 0.869 | 0.934 | 0.639 |
|  | Testing | 0.755 ［0.6234 - 0.8864］ | 0.705 | 0.610 | 0.900 | 0.926 | 0.529 |
| SVM | Training | 0.928 ［0.8786 - 0.9773］ | 0.912 | 0.910 | 0.918 | 0.963 | 0.812 |
|  | Testing | 0.776 ［0.6576 - 0.8936］ | 0.721 | 0.683 | 0.800 | 0.875 | 0.552 |
| KNN | Training | 0.911 ［0.8724 - 0.9490］ | 0.566 | 0 382 | 1.000 | 1.000 | 0.407 |
|  | Testing | 0.783 ［0.6526 - 0.9133］ | 0.656 | 0.585 | 0.800 | 0.857 | 0.485 |
| ExtraTrees | Training | 0.879 ［0.8305 - 0.9275］ | 0.810 | 0.826 | 0.770 | 0.895 | 0.653 |
|  | Testing | 0.702 ［0.5540 - 0.8497］ | 0.656 | 0.610 | 0.750 | 0.833 | 0.484 |
| MLP | Training | 0.930 ［0.8946 - 0.9659］ | 0.849 | 0.826 | 0.902 | 0.952 | 0.687 |
|  | Testing | 0.724 ［0.5848 - 0.8640］ | 0.656 | 0.537 | 0.900 | 0.917 | 0.486 |

*PPV*, positive predictive value; *NPV*, negative predictive value

Table S4 The receiver operating characteristic (ROC) curve difference between various Groups (Delong test).

| Groups | | 95% CI | *Z* | *P* |
| --- | --- | --- | --- | --- |
| Jounior1 | AI-assisted Jounior1 | 0.0191 - 0.2050 | 2.360 | 0.018 |
| Jounior2 | AI-assisted Jounior2 | 0.0285 - 0.2200 | 2.540 | 0.011 |
| Jounior3 | AI-assisted Jounior3 | 0.0034 - 0.1710 | 2.040 | 0.041 |
| Senior1 | AI-assisted Senior1 | -0.0240 - 0.0740 | 1.000 | 0.317 |
| Senior2 | AI-assisted Senior2 | -0.0240 - 0.0740 | 1.000 | 0.317 |
| Senior3 | AI-assisted Senior3 | -0.0240 - 0.0740 | 1.000 | 0.317 |
| AI-assisted Jounior1 | Senior1 | -0.0628 - 0.0872 | 0.319 | 0.750 |
|  | Senior2 | -0.0542 - 0.1030 | 0.609 | 0.543 |
|  | Senior3 | -0.0542 - 0.1030 | 0.609 | 0.543 |
| AI-assisted Jounior2 | Senior1 | -0.0768 - 0.1020 | 0.280 | 0.779 |
|  | Senior2 | -0.0920 - 0.0932 | 0.013 | 0.990 |
|  | Senior3 | -0.0920 - 0.0932 | 0.013 | 0.990 |
| AI-assisted Jounior3 | Senior1 | -0.0628 - 0.0872 | 0.319 | 0.750 |
|  | Senior2 | -0.0542 - 0.1030 | 0.609 | 0.543 |
|  | Senior3 | -0.0542 - 0.1030 | 0.609 | 0.543 |
